# Schwann cell-free adult canine olfactory ensheathing cell preparations from olfactory bulb and mucosa display differential migratory and neurite growth-promoting properties in vitro

**DOI:** 10.1186/1471-2202-14-141

**Published:** 2013-11-13

**Authors:** Frank Roloff, Susanne Ziege, Wolfgang Baumgärtner, Konstantin Wewetzer, Gerd Bicker

**Affiliations:** 1 Division of Cell Biology, University of Veterinary Medicine Hannover, Bischofsholer Damm 15/102, 30173 Hannover, Germany; 2 Department of Pathology, University of Veterinary Medicine Hannover, Bünteweg 17, 30559 Hannover, Germany; 3 Center for Systems Neuroscience Hannover, Hannover, Germany; 4 Department of Functional and Applied Anatomy, Hannover Medical School, Carl-Neuberg-Str.1, 30625 Hannover, Germany

**Keywords:** Glia, Scratch wound assay, Large animal model, Human NT-2 neurons, Regeneration

## Abstract

**Background:**

Transplantation of olfactory ensheathing cells (OEC) and Schwann cells (SC) is a promising therapeutic strategy to promote axonal growth and remyelination after spinal cord injury. Previous studies mainly focused on the rat model though results from primate and porcine models differed from those in the rat model. Interestingly, canine OECs show primate-like *in vitro* characteristics, such as absence of early senescence and abundance of stable p75^NTR^ expression indicating that this species represents a valuable translational species for further studies. So far, few investigations have tested different glial cell types within the same study under identical conditions. This makes it very difficult to evaluate contradictory or confirmatory findings reported in various studies. Moreover, potential contamination of OEC preparations with Schwann cells was difficult to exclude. Thus, it remains rather controversial whether the different glial types display distinct cellular properties.

**Results:**

Here, we established cultures of Schwann cell-free OECs from olfactory bulb (OB-OECs) and mucosa (OM-OECs) and compared them in assays to Schwann cells. These glial cultures were obtained from a canine large animal model and used for monitoring migration, phagocytosis and the effects on *in vitro* neurite growth. OB-OECs and Schwann cells migrated faster than OM-OECs in a scratch wound assay. Glial cell migration was not modulated by cGMP and cAMP signaling, but activating protein kinase C enhanced motility. All three glial cell types displayed phagocytic activity in a microbead assay. In co-cultures with of human model (NT2) neurons neurite growth was maximal on OB-OECs.

**Conclusions:**

These data provide evidence that OB- and OM-OECs display distinct migratory behavior and interaction with neurites. OB-OECs migrate faster and enhance neurite growth of human model neurons better than Schwann cells, suggesting distinct and inherent properties of these closely-related cell types. Future studies will have to address whether, and how, these cellular properties correlate with the *in vivo* behavior after transplantation.

## Background

Neurons of the mammalian central nervous system have a very restricted regenerative capacity in response to damage [1,2]. The potential of creating a favorable cellular environment for improving neurological recovery from traumatic spinal cord injury is currently a topic of intense basic and clinical research [3]. Transplantation of Schwann cells (SC) and olfactory ensheathing cells (OEC) appears to be a promising therapeutic strategy to facilitate axon regeneration and remyelination after spinal cord injury. During lifelong sensory neuron turnover, OECs continuously support axonal outgrowth from the periphery into the olfactory bulb of the CNS. Due to their potential to create a permissive environment for axon growth and accessibility by nasal biopsy, OECs derived from olfactory mucosa are considered to be compelling candidates for autologous cell grafts [4]. Indeed, in a recent study of Granger et al. on pet dogs with severe chronic spinal cord injury, intraspinal transplantations of OECs derived from the olfactory mucosal cultures caused an improvement in fore limb-hind limb coordination [5]. Several other transplantation studies have used OECs or Schwann cells in models of spinal cord injuries to restore myelination and promote axonal regeneration [6-9]. Grafting of cultured olfactory ensheathing cells from the olfactory bulb into the spinal cord promoted regrowth of lesioned long spinal axons [6-8,10].

Migration into and beyond the site of lesion is a critical point to bridge the glial scar for creation of a permissive environment over the whole lesion site. Early studies using Schwann cells from rat and mouse reported extensive migration of transplanted cells into the demyelinated regions of the lesion in the spinal cord [11,12]. Since the migratory properties of glial cell transplants contribute to the restoration of neuronal function in the injured CNS, we investigated the cellular motility of three purified glial types and evaluated whether motility could be up-regulated by application of cyclic nucleotide signaling molecules [13] and a phorbol ester.

To promote axonal regeneration transplanted cells can remove cellular debris of necrotic neurons and glia. Especially remaining myelin is a key factor of blocking axonal regeneration [14]. Both, OECs and Schwann cells are known to phagocytize bacteria as well as fragments of degraded neurons, however reports of phagocytosis of cellular debris after transplantation are still lacking [15-17]. Thus we studied whether OECs and Schwann cells can phagocytize microspheres in an *in vitro* co-culture system.

Another important feature of this study is the establishment of a Schwann cell-free preparation as reported [18]. The olfactory mucosa contains OECs and myelinating Schwann cells from trigeminal afferents and other non-myelinating cells. Moreover, the close phenotypic resemblance of OECs and Schwann cells and the expression of marker molecules such as the neurotrophin receptor p75 (p75^NTR^) and glial protein S100 represent obstacles for the selective identification and purification of pure OEC preparations that are free of Schwann cells. Using magnetic activated cell sorting, it has recently been shown that contaminating Schwann cells can be depleted from canine OEC preparations allowing further *in vitro* characterization of purified OECs from olfactory bulb (OB-OECs), olfactory mucosa (OM-OECs), and Schwann cells from fibular nerve [18].

To advance our understanding how these various groups of glial cells may facilitate axonal regeneration in the damaged CNS various *in vitro* assays were performed. Since a permissive environment created by transplants of migratory glial cells contributes to axonal outgrowth in the injured CNS, initially we investigated the cellular motility of the purified three glial types. To compare cell motility, a scratch migration assay which measures cell migration during the closure of a “wound” that is scratched into a confluent cell monolayer was used. In addition, it was investigated whether motility could be up-regulated by chemical manipulation of intracellular signaling cascades. So far, we found no evidence that glial migration is influenced by application of cGMP or cAMP signaling molecules [19,20], but activating PKC enhances motility. Glial cells may aid repair processes in the CNS by clearing cellular debris via phagocytosis. Using a phagocytosis assay, we demonstrated internalization of fluorescent microspheres into all three glial cell types.

Finally, glial cells were analyzed for their potential to improve neurite outgrowth in a co-culture system with human NT2 model neurons. These neurons were derived from the Ntera2/D1 clone of a well characterized teratocarcinoma cell line, which can be induced to differentiate into fully functional post mitotic neurons by retinoic acid treatment. NT2 cells resemble human embryonic stem cells [21] and the differentiation of NT2 cells into neurons has been suggested to mimic aspects of vertebrate neurogenesis [22-25]. The co-culture assays using OECs and SCs represent a needed prerequisite to evaluate the potential therapeutic impact of the three glial cell types for repair of spinal cord injuries in a large animal translational model and their future clinical application.

## Results

### Scratch migration assay

**Figure 1:**
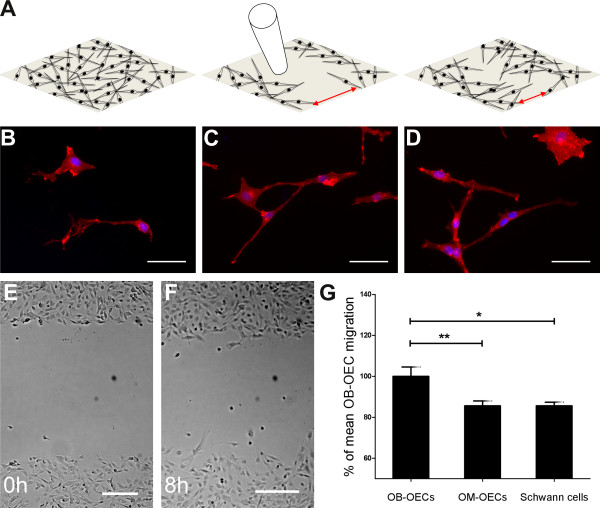
**Scratch wound assay to quantify and analyze migration rate of canine olfactory ensheathing and Schwann cells. (A)** Schematic drawing of the scratch wound assay. Confluent cell monolayer (left drawing) is scratched using a pipette tip (middle). The right drawing shows the closure of the wound. The gap width is photographed at t_0_ and t_8_ and measured using ImageJ (red arrows) **(B-D)** Representative images of OB-OECs **(B)**, OM-OECs **(C)** and Schwann cells **(C** stained for p75^NTR^ and DAPI are shown. All cells displayed either a spindle-shaped and process-bearing phenotype or a more flattened phenotype with only short processes emanating from the cell soma. **(E-F)** Representative images of the scratch at t_0_ and after 8 h migration are shown exemplarily for OB-OECs. **(G)** Migration rate differs after 8 h between cell types. OB-OECs showed significant higher migration rates than OM-OECs or Schwann cells whereas OM-OECs and Schwann cells showed no significant difference. Histograms present mean ± SEM of 7 (60 scratches, OB-OECs), 7 (48 scratches, OM-OECs) and 5 experiments (46 scratches, SCs). Statistical analysis was performed using an ANOVA and the Newman-Keuls *post-hoc* test (**p < 0.01, *p < 0.05). Cells are stained against p75^NTR^ (red) and DAPI (blue). Scale bars are 50 μm **(B-D)** and 200 μm **(E and F)**.

One therapeutic aspect of OEC cell transplantation for treatment of SCI is related to the glial ability to migrate within the injury site and to accompany regenerating neurites. To compare the motility of the purified canine glial cells, we used a scratch migration assay which tracks cell migration during the closure of a “wound” that is scratched into a confluent cell monolayer (Figure 1A) [26,27]. Immunocytochemical staining of purified cultures confirmed p75 neurotrophin receptor (p75^NTR^) expression in all types of glial cells (Figure 1B-D). High magnification images depicted a patchy appearance of immunoreactivity on the glial cell surface, indicative of selective membrane incorporation under cell culture conditions. We seeded the cells into 24-well-plates and performed a scratch wound to the confluent cell monolayer using a pipette tip (Figure 1A). Figures 1E and F show how a scratch wound in a confluent layer of purified OECs from the olfactory bulb induces glial migratory behaviour. The closure of the gap was monitored by following the advancement of the cell front over 8 h. Because this time interval is too short for significant cell proliferation, presence of cells in the gap largely reflects migration (Figure 1F). To estimate cell proliferation in the scratch wound, we performed a BrdU assay [28] and found less than 20% BrdU-positive cells at 8 h of incubation.

All three glial cell types displayed migratory capability and initiated wound closure. In the scratch assay, OB-OECs (n: 60) migrate considerably faster than OM-OECs (n: 58, 85.62% ± 2.337 of OB-OECs) and Schwann cells (n: 46, 85.62% ± 1.733 of OB-OECs, Figure 1G).

### Chemical manipulation of ensheathing cell migration

**Figure 2:**
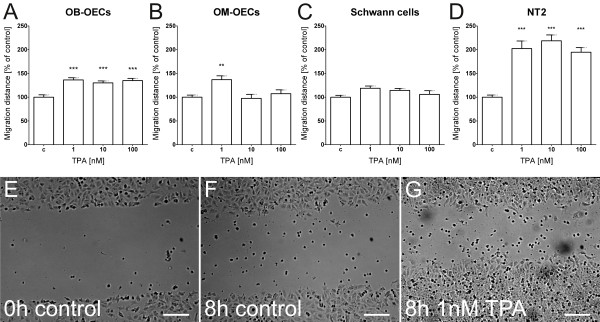
**Protein kinase C activator TPA enhances cell migration. (A)** OB-OECs showed an increase of migration to 130% compared to controls at 1 to 100 nM TPA in the scratch wound assay **(B)** Application of protein kinase C activator TPA to OM-OECs resulted in a significant increase to 144% compared to controls at 1 nM TPA, however, no difference was observed at 10 and 100 nM **(C)** Schwann cells showed no migratory effect after TPA treatment **(D)** NT2 precursor treated with TPA resulted in an increase of migration to approximately 200% compared to controls. **(E-G)** Human NT2 precursor migration increased when treated with the PKC activator TPA **(G)** compared to the control **(F)**. Histograms present mean ± SEM of 3 (Schwann cells, OB-OECs and OM-OECs) and 5 experiments (NT2 precursor). Statistical analysis was performed using an ANOVA and the Newman-Keuls *post-hoc* test (***p < 0.001, **p < 0.01). Scale bar is 200 μm.

Protein kinases C have been identified as essential regulators of migration in several cancer and epithelial cell types as well as for human neural progenitors [29-32]. In our motility assay, application of 1 nM of the protein kinase C activator TPA (12-O-tetradecanoylphorbol-13-acetate) resulted in a significant increase of cell migration for ensheathing cells from the olfactory bulb (136% of control, Figure 2A) and the olfactory mucosa (144% of control, Figure 2B). Raising the concentration to10 nM and 100 nM resulted in a still significant increased migration to 130% and 135% of control, respectively (Figure 2A). However, migration rates of OM-OECs fall back to control level at concentrations above 1 nM TPA. Schwann cells were less responsive to the PKC activator, showing no significant increase in cell migration (Figure 2C). As additional migratory cell type we used human NT2 precursor cells as positive control, representing an internal laboratory standard with known reactions to application of cyclic nucleotides and activation of the PKC pathway [28]. At concentrations of 1 to 100 nM of TPA (Figure 2D), these cells showed a two fold increase of their cell migration velocity.

**Figure 3:**
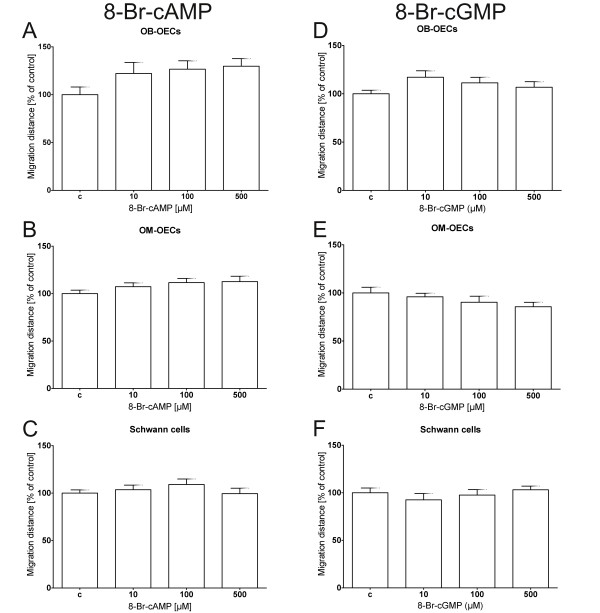
**Membrane permeable analogues 8-Br-cAMP and 8-Br-cGMP do not increase glial cell migration in a scratch wound assay. (A-C)** Incubation of canine olfactory ensheathing cells and Schwann cells with 8-Br-cAMP **(D-F)** or 8-Br-cGMP showed no significant increase of glial migration rate after 8 hours. **(A)** Ensheathing cells from the olfactory bulb showed a slight increase to 125% of control at 10 μM to 500 μM 8-Br-cAMP. However, this effect was not significant. **(B-C)** OM-OECs and Schwann cells showed no increase under cAMP treatment. **(D-F)** Ensheathing cells from the olfactory bulb showed a slight, not statistically significant increase to 120% compared to controls, at 10 μM 8-Br-cGMP whereas OM-OECs and Schwann cells showed no change in migration rate under cGMP treatment. Histograms present means ± SEM of 4 separate experiments (all cell types for 8-Br-cAMP) and 4 (OB-OECs, OM-OECs) and 3 (Schwann cells) for 8-Br-cGMP. Statistical analysis was performed using an ANOVA and the Newman-Keuls *post-hoc* test.

Dynamic regulation of intracellular cyclic nucleotide levels plays a key role in modulating cellular motility and regeneration in the nervous system [19,20]. Additionally Windus et al. reported that OEC motility regulates activity of pioneer growth cones and therefore neurite elongation in a positive manner [33]. To explore a potential involvement of cAMP and cGMP signal transduction in glial motility, we tested whether membrane permeable nucleotide analogues might facilitate the closure of the gap. For this purpose we seeded the cells into 24-well-plates and performed a scratch wound to the confluent cell monolayer using a pipette tip (Figure 1A). Both pharmacological agents were applied to the scratch wound assay using concentrations of up to 500 μM of the membrane permeable cAMP analogue 8-Br-cAMP (Figure 3). Migration rates of OB-OECs increased slightly to 122% and 127% of control at 10 μM and 100 μM, respectively (Figure 3A). However, this increase was not statistically significant. For Schwann cells and OM-OECs no increase of migration rates could be observed (Figures 3B and C). Even the highest concentration of 500 μM 8-Br-cAMP had no stimulatory effect on cell migration. Next we tested the cGMP/PKG pathway which has been shown to stimulate NT2 cell migration [28] (Figure 3D-F). Application of the cGMP analogue 8-Br-cGMP had no significant effects on cell migration in all three glial cultures at concentrations from 10 to 500 μM.

**Figure 4:**
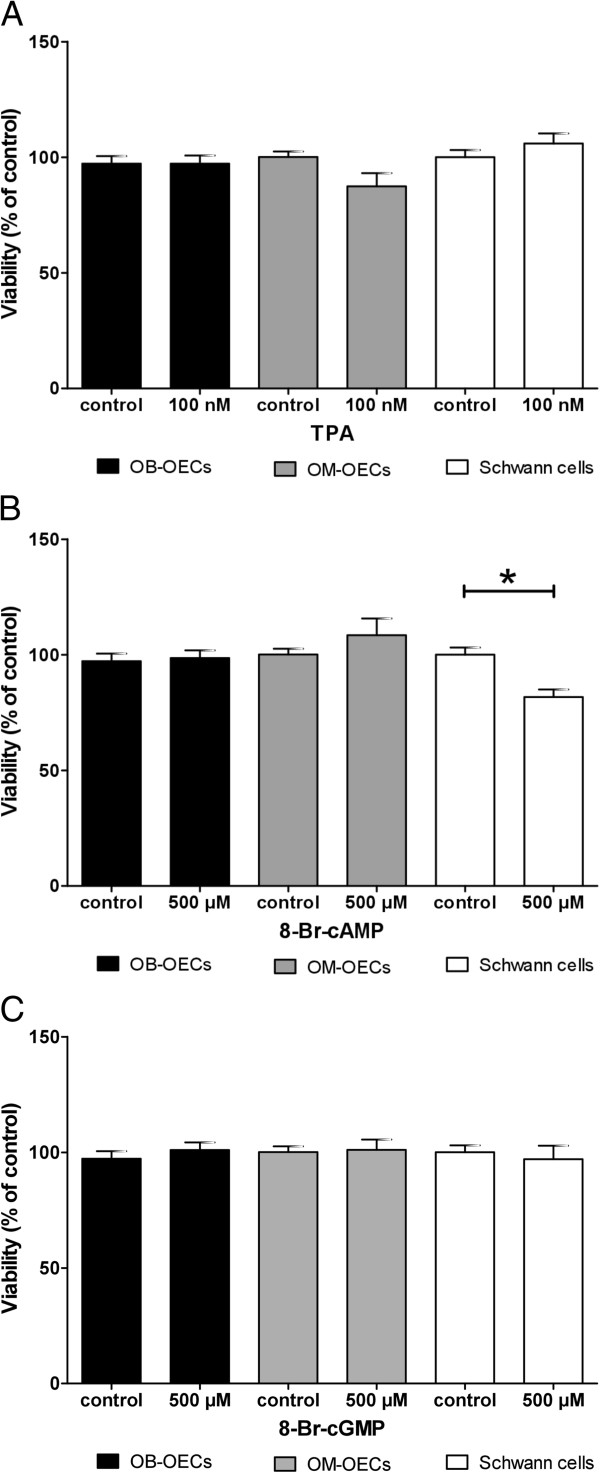
**Influence of nucleotide analogues 8-Br-cAMP, 8-Br-cGMP, and the PKC activator TPA on glial cell viability. (A)** The highest concentration of 500 μM of the cGMP analogue 8-Br-cGMP had no significant effect on cell viability of olfactory ensheathing cells and Schwann cells. Schwann cell showed a slightly decreased viability (86% of control) which was not significant. **(B)** OB-OECs and OM-OECs showed no significant difference between viability of controls at a concentration of 500 μM 8-Br-cAMP. Schwann cells showed a significant decrease in cell viability of 81% compared to controls. **(C)** Cell viability of all three cell types was not significantly decreased at 100nM TPA treatment in comparison with control. Histograms present the means ± SEM of 3 separate experiments with at least three wells per experiment. Statistical analysis was performed using an ANOVA and the Newman-Keuls *post-hoc* test (*p < 0.05).

Treatment of glial cell cultures with all chemical agents including the cell permeable nucleotide analogues 8-Br-cGMP and 8-Br-cAMP or the PKC activator TPA, did not affect cell viability, even at the highest applied concentration (Figure 4).

### Phagocytic activity of OECs

**Figure 5:**
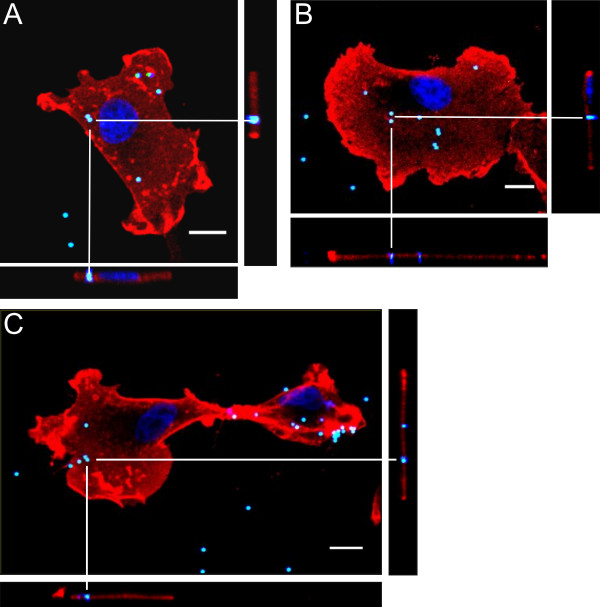
**Glial phagocytosis of microspheres.** Canine glia showed phagocytosis of microspheres. OB-OECs **(A)**, OM-OECs **(B)** and SCs **(C)** contained 6 to 18 microspheres as indicated by confocal z-axis series analysis. XY, XZ, and YZ projections revealed that the microspheres were taken up and engulfed by the canine glia. OB-OECs, OM-OECs and Schwann cells incubated 2 h at 4°C showed no signs of engulfed microspheres indicating that phagocytosis is absent at low temperature (data not shown). Cells are stained against p75^NTR^ (red) and DAPI (blue). YG microspheres appear cyan due to fluorescence in the blue and green spectrum. Scale bar is 10 μm.

Recent studies suggested that olfactory ensheathing cells may be the primary innate immunocytes in the olfactory pathway [16,17,34]. To obtain evidence for phagocytic activity of the canine glial cells, we examined whether they could internalize YG fluorescent microspheres of 1 μm diameter. After addition of microspheres to glial cells seeded on poly-L-lysine coated chamber slides, phagocytosis could be observed for OB-OECs (Figure 5A), OM-OECs (Figure 5B) and Schwann cells within two hours of cell culture (Figure 5C). All cells showed at least six microspheres located in the cytoplasm of the cells, confirmed by confocal z-stacks. Glial cells incubated in 4°C cold culture medium showed changes in their morphological appearance and did not internalize the microspheres. Cells cultured at 4°C failed to engulf microspheres (data not shown). To investigate how the distinct glial cell types would affect healthy neurons, we prepared co-cultures with cell tracker green CMFDA labeled human NT2 neurons (Invitrogen, Karlsruhe, Germany). Under normal cell culture conditions, all of the different canine glial cells showed no evidence for phagocytosis and engulfment of neurons (data not shown).

### Canine ensheathing cells of the bulb modulate neurite outgrowth of developing human neurons

To analyze potential interactions between the isolated olfactory ensheathing and Schwann cells with neurons, we measured how canine glial cells influence parameters of neurite outgrowth. We co-cultured developing human NT-2 neurons after two weeks of retinoic acid treatment (2wk RA) [35] together with ensheathing cells from the olfactory bulb (OB-OECs), the olfactory mucosa (OM-OECs) and Schwann cells from the fibular nerve. Outgrowing neurites of the developing neurons were visualized by immunofluorescence, using an antibody against the neuronal cytoskeletal marker β-III-tubulin.

**Figure 6:**
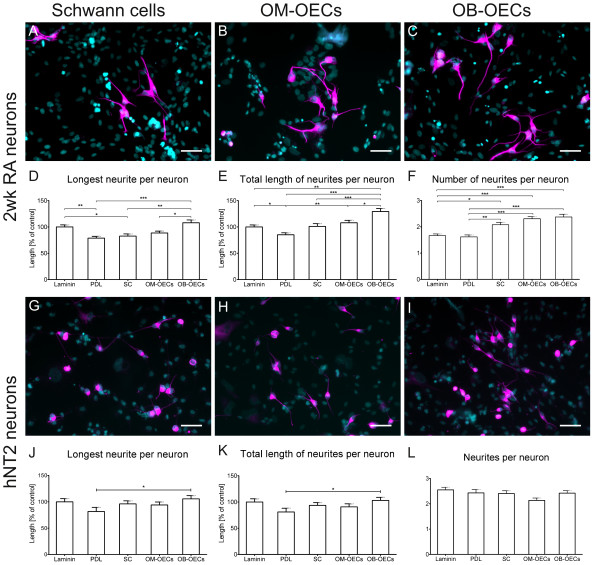
**Olfactory ensheathing cells promote neurite outgrowth of developing, but not mature NT2 neurons.** Human developing neurons (2wk RA) were co-cultured with Schwann cells **(A)** and ensheathing cells from the olfactory mucosa **(B)** and the olfactory bulb **(C)** for 24 h. **(D)** OB-OECs showed a permissive effect for elongation of the longest neurite compared with PDL, Schwann cells and OM-OECs but not with laminin. **(E)** Neurite outgrowth on OB-OECs resulted in a significant increase of total length compared with all other substrates and cell types. **(F)** Number of neurites is increased for neurons seeded to Schwann cells, OM-OECs and OB-OECs but not for neurons seeded to a laminin or PDL coating. **(G-I)** Mature neurons grown on canine Schwann cells **(G)**, mucosal **(H)** and bulbar ensheathing **(I)** cells. **(J)** Elongation of the major neurite was not significantly increased when seeded to canine glia **(K)** Total length of neurites was neither affected by glial cells when compared with control **(L)** Number of neurites appeared slightly, but not significantly reduced for neurons grown on OM-OECs. Cells were fixed and stained for β-III-Tubulin (magenta) and DAPI (cyan) after 24 h of co-culturing. Histograms present the means ± SEM of 4–5 experiments (2wk RA) and 2–3 experiments (hNT2). Statistical analysis was performed using Kruskal-Wallis-test (***p < 0.001, **p < 0.01, *p < 0.05). Scale bar is 50 μm.

Seeding of 2wk RA neurons to laminin, PDL coated culture wells, and a confluent layer of canine glia resulted in the growth of long processes. These extending neurites stained for β-III-tubulin, while olfactory ensheathing (OB-OECs, OM-OECs) and Schwann cells did not express immunoreactivity (Figures 6A-C). After 24 h outgrowth most of the neurons showed one long major neurite and up to five shorter processes emanating from the cell body. Neurons on PDL extended a shorter major neurite compared to neurons plated on laminin. Outgrowth on Schwann cells resulted in similar major neurite lengths as for neurons on a PDL coating which was, however, significantly shorter than for neurons on laminin only (Figures 6A and D). The longest major neurite could be observed for neurons cultured on OB-OECs. However, the neurite extension was not significantly larger than on the laminin coating alone (Figure 6D).

Next we looked at the total length of all neurites per neuron and found a significantly enhanced total length for neurons grown on laminin compared to PDL (Figure 6E). Developing 2wk RA neurons seeded to Schwann cells and OM-OECs showed the same total length of neurites compared to neurons grown on Laminin. The use of OB-OECs as substrate for neurite outgrowth resulted in the largest effect on total length with an increase of up to 129% compared to laminin-coated control. The overall neurite length was significantly larger than for neurons grown on PDL, laminin, Schwann cells or ensheathing cells from the olfactory mucosa (OM-OECs, Figure 6D and E). Next we focused on the initiation and outgrowth of primary neurites. Culturing the 2wk RA neurons on canine glia resulted in an increase in neurite number for Schwann cells (p < 0.05), OM-OECs and OB-OECs (both, p < 0.001) compared to controls on laminin or PDL (Figure 6A-C and F).

To reveal age-dependent neuron intrinsic mechanisms that might affect neurite formation, we repeated the same experiments with mature human neurons (hNT2). There was indeed a remarkable difference compared to the results of the developing 2wk RA neurons. Evaluating the longest neurite per neuron, we could only detect a significant effect between outgrowth on PDL (Figure 6J, 82% of control) and outgrowth on OB-OECs with 105% of control (Figure 6I). Neither outgrowth on Schwann cells (96% of control) nor on OM-OECs (94% of control) resulted in an elongated major neurite compared with outgrowth on a laminin coating only (Figure 6J). Next we analyzed the total neurite length of all neurites per neuron for all five outgrowth substrates. Outgrowth on PDL (82%), SCs (94%) and OM-OECs (91%) resulted in a not significant reduction of neurite lengths (Figure 6K). Neurons grown on OB-OECs formed significant longer neurites compared with PDL, but not with the laminin coating (Figure 6I and K). To check whether this elongated neurite length is due to the increase in the number of processes, we counted for each substrate the number of neurites per neuron. NT2 neurons grown on laminin formed on an average 2.6 neurites per neuron whereas PDL coating resulted in 2.4 neurites (Figure 6). Overall, there was no significant difference in neurite formation between neurons grown on laminin or PDL compared to co-culture with Schwann cells (2.4), OM-OECs (2.1) and OB-OECs (2.4).

## Discussion

### Assays for cellular functions of purified canine OECs

Olfactory ensheathing cells have claimed to be a unique cell type according to their special localization in the PNS and CNS as well as their special morphology known for their ability to bridge the gap between the central nervous (CNS) and the peripheral nervous system (PNS) in the mammalian olfactory neuraxis [36-38]. Several studies have reported beneficial effects on neuronal regeneration after OEC transplantation into animals with different lesion paradigms in the PNS [39-41] and CNS [5,42-44]. Except for a few studies, the majority of investigations concerning the regenerative potential of ensheathing glia have been performed in rodent models [9,45-47]. Only a few studies used canine or porcine ensheathing cells as translational model or even human ensheathing cells to show differences to rodents [4,5,48-51].The present study is one of the first to utilize a comparative approach for analysis of the migratory, phagocytic and neurite growth-promoting properties of purified Schwann cell-free preparations of OECs and Schwann obtained from a large animal model [18].

### Olfactory ensheathing cell and Schwann cell migration

To test for differences in migratory capabilities, we performed a scratch wound assay over the rather short time interval of 8 hours. Therapeutic treatment of SCI clearly would require longer migration times in the range of weeks. However, the rather short migration time of 8 h is a concession to the simplicity of the in vitro test system. We have performed a cell proliferation assay and found less than 20% BrdU-positive cells at 8 h of incubation. A significant effect of proliferation on the motility component of the scratch wound assay could therefore be excluded. Comparative analysis revealed a striking difference in migratory capacities of OB-OECs, OM-OECs and SCs. Former studies have reported either a difference in migration rates between OECs and Schwann cells in the X-radiated spinal cord [52] or no difference between these cells after transplantation in a spinal cord injury model [53]. The occurrence of lamellipodial waves and the density of cells are reported to influence the rate of *in vitro* migration [54]. However, most of the studies agree that migration within the injured spinal cord is essential to establish a permissive environment to promote sprouting and inhibit scar formation [45,55-57]. So far, *in vitro* migration rates of SC-free cultures of OECs obtained from the olfactory bulb and mucosa have not been compared to Schwann cells in a scratch wound assay. This study shows that SC-free cultures of canine OECs from the olfactory bulb migrate faster than OECs from the mucosa or Schwann cells from the tibial nerve (Figure 1G). Moreover, activation of the protein kinase C by phorbol ester treatment significantly promoted migration of canine OECs. This was also found for the human NT2 cell line. The absence of a response to phorbol ester treatment in canine Schwann cells indicated a differential expression of signal transduction pathways regulating migratory behavior in ensheathing compared to peripheral glial cells. A former study of Simón et al. reported that inhibiting PKC resulted in a decreased expression of PAI-1 in human OECs associated with a reduced OEC-dependent regeneration of axons of adult retinal ganglion cells [58].

Rodent OECs showed an increase in proliferation, expression of GFAP and a morphology change of OECs upon the cAMP-elevating agent Forskolin, whereas primate OECs lacked such a response [48].

Activation of the cAMP/PKA pathway overcomes the regeneration inhibitory effects of myelin *in vitro* and neuritogenesis of human NT2 neurons is elevated after treatment with cAMP [35]. Therefore, to improve ensheathing glia migration we applied 8-Br-cAMP [35,59]. Though Wang and Huang and Vincent et al. showed a change of the glia morphology from an astrocyte-like to a Schwann cell-like cell type under elevated levels of cAMP, we could not observe any morphological difference to cells cultured under control conditions [60,61]. In line with this observation, culturing canine glia with 8-Br-cAMP did not change the glial cell migration rate (Figure 3A-C). Culturing OECs in the presence of low concentrations or the absence of serum resulted in a shift from a flattened to a more spindle-shaped cell morphology [61]. The absence of cAMP-mediated effects for canine OECs was already shown by Techangamsuwan et al. and supports observed differences in migratory properties of human and canine OECs compared to rodent OECs [48].

Similarly, the nucleotide cGMP, known to facilitate migration of human NT2 neurons, fetal human neural progenitor cells and human bronchial epithelial cells [28,62,63] did not enhance canine glia migration (Figure 4).

### Phagocytic activity of ensheathing glia

During normal olfactory neuron turnover, OECs remove debris of dead receptor cells by phagocytosis [15,17]. Moreover, recent studies showed, that OECs and Schwann cells represent the first defence mechanism of the nasal cavity and they react upon contact to bacteria [16,34,64]. Along with this function, activation of OECs with lipopolysaccharides (LPS) resulted in a strongly increased phagocytic activity consisting of ingestion of apoptotic cell fragments *in vitro* and in vivo. The remains of degraded olfactory receptor neurons (ORNs) are known to block axonal regeneration and outgrowth of new ORNs in general [65,66]. In the nasal mucosa, apoptotic ORNs and cell debris like O4^+^ fragments are engulfed by OECs and removed from the olfactory pathway [15]. OECs injected into the subretinal space in a retinitis pigmentosa model [67] or a demyelinating spinal cord model of rats, cleaned the subretinal space from accumulated debris or phagocytized degenerated host tissue. This has also been found in intracranial sections of rat olfactory bulbs [68].

Culturing both canine OECs and Schwann cells with 1 μm microspheres resulted in engulfment and their localization into the cell within two hours (Figure 5). This could be prevented by slowing down the cell metabolism at 4°C. The phagocytosis of the microspheres by cultured canine glia resembles the removal of small cellular debris. In contrast, co-culturing of intact human NT2 neurons without any indications of apoptosis together with canine ensheathing glia for 24 hours provided no evidence for a phagocytic uptake of fluorescent neuronal material.

### Neuritogenesis of human model neurons in co-cultures

In the present study, we show for the first time, that canine ensheathing glia promote neurite outgrowth of developing human model neurons (Figure 6). Culturing developing NT2 neurons on a substrate of canine ensheathing glia from the PNS and CNS resulted in an increased sprouting and elongation of neurites. The positive interaction between canine cells and human model neurons is also in support of a concept using dogs as a translational model for the development of neural repair strategies in human patients [5,18,50,69].

Previously, we showed a preferential growth of dorsal root ganglion (DRG) neurons on canine OM-OEC, OB-OECs and Schwann cells [70]. Comparative analysis revealed that neurite length of DRG neurons and numbers of branching points were significantly increased when co-cultured with OM-OECs instead of OB-OECs and Schwann cells, respectively. Nevertheless, DRG neurons growth on OB-OECs and Schwann cells was improved compared to OM-OECs. DRG neurons cultured together with OM-OECs tended to stick to the poly-L-lysine substrate more firmly than to the cell surface of the glial cells. Using differentiating NT2 neurons we obtained an overall beneficial effect of canine OB-OECs, OM-OECs and SCs compared to a poly-D-lysine or the permissive laminin control. The number of neurites was higher in co-cultures of canine glia compared to both poly-D-lysin and laminin controls. These findings indicate that canine glia support the initiation of primary neurites and the elongation of these neurites. Due to the rather confluent glia cell layer, we could not directly evaluate whether co-cultured human NT2 neurons were directly associated to canine glia or the underlying substrate. Thus we cannot distinguish whether the growth promoting effects are due to soluble factors or direct contact to the glial cell surface.

The results obtained from NT2 neurons differ from results obtained after co-culturing rat DRG neurons with the same canine glial cells in a previous study [70]. DRG neurons are a unique type of neurons projecting two axonal branches; therefore reported differences could be neuron specific. Dorsal root ganglion neurons exhibit a centrally projecting branch extending into the spinal cord and the peripheral branch projecting through a peripheral nerve [71]. DRG neurons showed regeneration when the peripheral branch was lesioned first, but failed to regenerate when the central branch was lesioned [72]. Fudge and Mearow reported that a dissociated DRG culture is a heterogeneous mixture of different neuron phenotypes showing various responses to a permissive environment [73]. The close resemblance of the DRG entry zone and the olfactory bulb, both displaying a bridge between PNS and CNS, encouraged Ramón-Cueto and Nieto-Sampedro to use DRG neurons as a “proof of principle” for the regeneration facilitating effects of OECs [74]. In contrast to DRG neurons, human model neurons of the present study resembled more closely the CNS phenotype [35,75-77] which might account for the neuron specific differences between this and our former study [70].

The regenerative capacity of neurons is strongly reduced with age [78]. To account for this neuron-intrinsic limitation of neurite regeneration, we performed the co-culture experiments with NT2 neurons of mature developmental stage [75,76]. There was indeed a remarkable difference to the results of the developing 2wk RA neurons (Figure 6). Using mature neurons, there was only a weak increase between outgrowth on OB-OECs and outgrowth on PDL control and no positive effect on neurite formation for the other glial co-culture conditions. The reduction in regeneration between the 2wk RA and the mature neurons on glial co-cultures suggested that NT2 model neurons can mimic the loss in regenerative capacity that is generally observed in experimental animal models [1,2,79,80]. This does not necessarily imply that glial transplantations into mature neural tissue are ineffective to treat spinal injury. The reported beneficial effects of OEC transplantations into the injured spinal cord might result from clearance of cellular debris, axon guidance by migrating OECs, and the formation of tunnel-shaped pathways for regenerating axons [10,45,52,81,82]. In summary, co-cultures of canine glia and NT2 neurons appear to mimic cellular mechanisms of neural regeneration in damaged nerve tissue, comprising a useful platform for screening novel neuroprotective agents.

## Conclusions

This *in vitro* study supports the therapeutic approach that OEC and Schwann cell transplantation into the spinal cord generates a permissive environment for regeneration in the central nervous system. We demonstrate differences of ensheathing glial cell types with respect to migration and neurite outgrowth from neurons. OECs from the olfactory bulb migrate faster and enhance neurite growth of human model neurons better than OECs from the mucosa and Schwann cells. We challenged various signal transduction pathways with enzyme activators and show that stimulation of the protein kinase C pathway enhances migration of OECs from the olfactory bulb. Moreover, we demonstrate for the first time, that canine ensheathing glia promote neurite outgrowth of developing human model neurons.

## Methods

All test substances were diluted in Dulbecco’s modified Eagle medium (canine glia, DMEM Gibco-Invitrogen, Karlsruhe, Germany) or DMEM/F12 (NT2 cells, Gibco-Invitrogen) containing 10% fetal bovine serum (Invitrogen), 1% penicillin and streptomycin. The cAMP analogue 8-Br-cAMP (8-Bromoadenosine 3′,5′-cyclic monophosphate), the cGMP analogue 8-Br-cGMP (8-Bromoguanosine 3′,5′-cyclic monophosphate) were purchased from Sigma (Taufkirchen, Germany). The protein kinase C activator 12-*O*-tetradecanoylphorbol-13-acetate purchased from Alomone Labs (Jerusalem, Israel). Unless stated otherwise, all chemicals were obtained from Sigma.

### Cell cultures

All animals used for cell isolation were treated according to the legal and ethical requirements of the University of Veterinary Medicine Hannover (Germany). The procedures complied with the guidelines of animal welfare as laid down by the German Research Council (DFG). Purified primary OEC and Schwann cell cultures were generated as described previously [18].

OM-OEC cultures were isolated from the caudal regions of the nasal septum and conchae. All Schwann cells were removed immediately from primary cell suspensions by using anti-HNK-1 (OM-OECs) and anti-p75^NTR^ antibodies (OM-OECs, OB.OECs) and magnet-activated cell sorting (MACS; Miltenyi Biotec, Bergisch Gladbach, Germany). Schwann cells were obtained from the fibular nerve after removal of the epineural sheath [83]. Trypsin, hyaluronidase type IV, and collagenase type XI (0.5% each, Sigma Aldrich, Taufkirchen, Germany) were used to digest nerve preparations after 5–7 days of pre-degeneration *in vitro* by incubating nerve fibers [18]. Canine glia were cultured in T75 culture flasks with DMEM containing additionally 20 ng/ml recombinant human FGF-basic (fibroblast growth factor, Peprotech). Cells were trypsinized (Trypsin-EDTA, PAA, Marburg, Germany) twice a week and transferred to new T75 flasks and cultured under standard conditions (37°C, 5% CO_2_). Purity of glia cell cultures was about 95% as earlier reported in Ziege et al. [18]. NT2/D1 precursor cells (NT2) were purchased from the American Type Culture Collection (ATTC, Manassas, VA20108, USA). Neuronal differentiation was done as previously described [75,76]. After grown to confluence NT2 precursor cells were trypsinized (Trypsin-EDTA, Gibco-Invitrogen) and cultured in 95 mm, bacteriological grade Petri dishes (Greiner, Hamburg, FRG) at a density of 5 × 10^6^ cells/dish in 10 ml Dulbecco’s modified Eagle medium (DMEM/F12,) supplemented with 10% fetal bovine serum, 1% penicillin and streptomycin and 10 μM retinoic acid to start neuronal differentiation. After 7–10 days, cells were trypsinized again and transferred to T75 culture flasks with retinoic acid at a density of 4 × 10^7^ cells / flask. After additional 7–10 days, cells were trypsinized and cultured 2 days with DMEM/F12 until they were transferred to T175 flasks with DMEM/F12 containing mitotic inhibitors (1 μM 1-6-D-arabinofuranosylcytosine, 10 μM 2′-deoxy-5-fluorouridine and 10 μM 1-β-D-ribofuranosyluracil). During culturing of cells with medium containing mitotic inhibitors, neurons became visible after 7–10 days. Neurons were selectively trypsinized, counted and used in up-coming experiments. Neurons termed as 2wk RA representing a model for developing human neurons were cultured for additional 7 days with 10 μM retinoic acid in petri dishes instead of being transferred to T175 flasks with inhibitor medium [35]. After two weeks of retinoic acid treatment in petri dishes cells were dispersed, counted and used for neurite outgrowth experiments.For all cell culture experiments, canine glia of passage 7 (OB-OECs, OM-OECs and SCs), 2wkRA neurons of passage 20–30 and hNT2 passage 22–31 were used.

### Scratch wound assay

Migration of glial cells (passage 7) and human NT2 precursor (passage 22–30) cells was measured by the scratch wound assay as described by Liang et al. [27]. Olfactory ensheathing cells, Schwann cells and NT2 precursor were plated at a density of 150.000 -200.000 cells to poly-L-lysine coated (glia) and uncoated (NT2 precursor) 24-well-plates (Corning Costar, Kaiserslautern, Germany) 24 hours prior to the scratch. The next day a cell free area spanning approximately 600 μm in diameter was scratched using a crystal 10 μl pipette tip (Figure 1A). The wells were washed with medium to remove cellular debris. Cells were incubated with DMEM (canine glia) or DMEM/F12 (NT2) containing the cGMP analogue 8-Br-cGMP (10, 100, 500 μM), the cAMP analogue 8-Br-cAMP (10, 100, 500 μM) and PKC activator phorbol-12-myristate 13-acetate (TPA, 1, 10, 100 nM). The scratch was photographed 0 h and 8 h after its generation using a Zeiss Axiovert 200 microscope equipped with a Cool Snap camera (Photometrics, Tucson, Ariz., USA) and Meta Morph software (Molecular Devices, Sunnyvale, Calif., USA). Gap width was measured using ImageJ 1.46d (NIH, http://rsbweb.nih.gov/ij/, USA).

The distance was calculated by subtracting the average gap width after 8 h from the average gap width at 0 h divided by 2 (Figures 1E and F). BrdU incubation caused the labelling of only a few cells in the scratch wound (data not shown). Due to the short time interval of 8 h, we can rule out a significant contribution of cell proliferation to the closure of the gap. Contrast enhancement and image overlay was done with ImageJ.

### Phagocytosis assay

Phagocytic activity was determined for olfactory ensheathing cells and Schwann cells *in vitro* using latex microsphere beads. Glial cells were seeded to poly-L-lysine (100 μg/ml) coated Nunc® Lab-Tek® 8 Chamber Slides™ (Sigma-Aldrich, Taufkirchen, Germany) at a density of 30,000 cells per well. Cells were incubated at 37°C and 5% CO_2_ in DMEM medium containing 5% FBS. After 12 hours yellowgreen fluorescent fluoresbrite carboxylated microspheres (1 μm diameter, Polysciences Europe GmbH, Eppelheim, Germany) were coated with FBS for 60 minutes at room temperature before they were diluted with DMEM medium to a final concentration of 2.8×10^7^ microspheres/ well. After 60 to 120 minutes at 37°C in the incubator, cells were fixed with 4% PFA (paraformaldehyde) for 15 minutes at room temperature. Remaining microspheres were rinsed off with PBS and 0.1% Triton X-100. As a negative control glial cells were incubated at 4°C. Afterwards Chamber Slides™ were stained for p75^NTR^ and 4′,6-diamidino-2-phenylindole (DAPI, Invitrogen, Karlsruhe, Germany) to determine co-localization of YG microspheres and glial cells. Phagocytized microspheres were localized taking z-axis series of single cells on the Chamber Slides™, using a Leica TCS SP5 AOBS confocal microscope. Using the confocal series we created XY, XZ and YZ images to check whether the microspheres are located on the inside of the cell or on the exterior (Figure 5).

### Neurite outgrowth assay

To monitor effects of glial cells on neurite outgrowth of human neurons we seeded hNT2 (passage 22–31) neurons and 2wk RA (passage 23–30) neurons on different substrates. As control coatings we used poly-D-lysine (10 μg/ml) and poly-D-lysine/laminin (100 μg/ml). Glial cells of passage 7 were seeded 1 hour prior to neuron seeding at a density of 25,000 cells/well to poly-L-lysine coated 96-well-plates (Corning Costar, Kaiserslautern, Germany). After attachment of glial cells to the substrate, neurons were seeded at a density of 10,000 cell/well to the OB-OECs, OM-OECs and the Schwann cells with a 1:1 ratio of DMEM and DMEM/F12 medium. Neurons, cultured for 24 h under 37°C and 5% CO_2_, were fixed and stained for β-III-Tubulin to evaluate the neurite outgrowth. For each neuron all neurites were measured using ImageJ. Furthermore the longest neurite and number of extending processes for each neuron were determined. For evaluation of the effect on neurite outgrowth only neurons with an apparent neurite were taken into account. Each experiment was performed at least in six wells and repeated three to five times with at least 74 neurons (2wkRA neurons) and 50 neurons (hNT2 neurons), per experiment, respectively.

### Immunocytochemistry

Immunocytochemistry was performed on mature hNT2 neurons and dispersed 2wk RA neurons as previously described [28]. Human neurons were fixed with 4% PFA and permeabilized with 0.1% Triton X-100. The monoclonal antibody β-III-Tubulin (1:10000, Sigma, Taufkirchen, Germany) was applied overnight at 4°C. The biotinylated secondary antibody (Vector, Burlingame, Mass., USA) was applied for at least 1 h at room temperature before Streptavidin coupled Cy3 was added for 1 h at room temperature to detect immunofluorescence. Nuclei were visualized using DAPI (4′6-diamidino-2′henylindoldihydrochloride, 0.1 μg/ml, Sigma, Taufkirchen, Germany) as counterstain.

### Statistical analysis

Graph Pad Prism was used for statistical evaluation. Data are expressed as the mean ± SEM. Significant differences were determined by one-way-ANOVA and the Newman-Keuls Multiple Comparison Test (scratch wound assay) or the Kruskal-Wallis Test and the Dunns Test (neurite outgrowth assay). Quantitative results are usually based on 3–5 independent experiments performed at least in triplicate. Significant levels are: * < 0.05, ** < 0.01, *** < 0.001.

## Authors’ contributions

FR, SZ, WB, KW, and GB conceived and designed the experiments. FR performed the cell cultures, immunocytochemistry, quantitative assays and generated the figures. SZ purified the distinct canine glial cell populations. FR and GB analyzed the data. FR, WB, KW, and GB wrote the manuscript. All authors read and approved the final manuscript.
